# Pediatric bicycle-related head injuries: a population-based study in a county without a helmet law

**DOI:** 10.1186/s40621-015-0048-1

**Published:** 2015-07-07

**Authors:** Ruchi Kaushik, Isabelle M Krisch, Darrell R Schroeder, Randall Flick, Michael E Nemergut

**Affiliations:** Mayo Clinic Children’s Center, 200 First Street SW, Mayo 16E, Rochester, MN 55905 USA

**Keywords:** Bicycle helmets, Head injuries, Public health

## Abstract

**Background:**

Head injuries are the leading cause of death among cyclists, 85 % of which can be prevented by wearing a bicycle helmet. This study aims to estimate the incidence of pediatric bicycle-related injuries in Olmsted County and assess differences in injuries between those wearing helmets vs. not.

**Methods:**

Olmsted County, Minnesota residents 5 to 18 years of age with a diagnostic code consistent with an injury associated with the use of a bicycle between January 1, 2002, and December 31, 2011, were identified. Incidence rates were calculated and standardized to the age and sex distribution of the 2000 US white population. Type of injuries, the percentage requiring head CT or X-ray, and hospitalization were compared using a chi-square test. Pediatric intensive care unit (PICU) admission, permanent neurologic injury, seizure, need for mechanical ventilation, and mortality were compared using Fisher’s exact test.

**Results:**

A total of 1189 bicycle injuries were identified. The overall age-adjusted incidence rate of all injuries was 278 (95 % CI, 249 to 306) per 100,000 person-years for females and 589 (95 % CI, 549 to 629) for males. The corresponding rates for head injuries were 104 (95 % CI, 87 to 121) for females and 255 (95 % CI, 229 to 281) for males. Of patients with head injuries, 17.4 % were documented to have been wearing a helmet, 44.8 % were documented as not wearing a helmet, and 37.8 % had no helmet use documentation. Patients with a head injury who were documented as not wearing a helmet were significantly more likely to undergo imaging of the head (32.1 percent vs. 11.5 %; *p* < 0.001) and to experience a brain injury (28.1 vs. 13.8 %; *p* = 0.008).

**Conclusions:**

Children and adolescents continue to ride bicycles without wearing helmets, resulting in severe head and facial injuries and mortality.

## Background

Unintentional injuries remain the leading cause of death among children and adolescents in the United States of America (USA) (Rezendes 2006). Bicycle riding is common among children and adolescents in the USA, providing recreation, transportation, and a pleasurable form of aerobic activity (Centers for Disease Control and Prevention 1999). However, apart from automobiles, bicycles are tied to more childhood injuries than any other consumer product (Safe Kids Worldwide 2014). In 2010, 112 children and adolescents died and over 200,000 children 5 to 14 years of age visited the emergency department (ED) for bicycle-related injuries (Centers for Disease Control and Prevention 1999; 2012). Furthermore, these injuries represent the third leading type of sports and recreation-related activity for which 15–19-year-old males visit the ED (Centers for Disease Control and Prevention 2013). Numerous risk factors for serious injury are associated with bicycling, including collision with a motor vehicle, speed greater than 15 miles per hour, alcohol use, male sex, and an unsafe riding environment (Rivara et al. 1997; Thompson and Rivara 2001; Li et al. 2001; Hoffman et al. 2010). It has been reported that approximately 75 % of all bicycle-related mortality is secondary to head injuries, 85 % of which could have been prevented by wearing a bicycle helmet (The Center for Head Injury Statistics 2014). While child cyclists, particularly younger children, appear to be at greater risk of head injuries than adults, helmet use is low despite legislation requiring helmet use among younger riders in 21 states and the District of Columbia, as well as the adoption of many local city and county ordinances (Coffman 2003; Insurance Institute for Highway Safety & Highway Loss Data Institute 2014). For example, among the 70 % of high school students nationwide who had ridden a bicycle in 2011, 87 % had rarely or never worn a bicycle helmet, a prevalence that has changed little from 2001 to 2011 (Coffman 2003).

The Cochrane Collaborative conducted an analysis of controlled studies evaluating the effect of bicycle helmets on injury prevention. The review found that helmets provide up to a 63 to 88 % reduction in the risk of head, brain, and severe brain injury for cyclists of all ages as well as a 65 % reduction of injuries to the upper- and mid-face (Thompson et al. 2000). This protection was sustained independent of the mechanism of bicycle injury (crashes involving motor vehicles vs. crashes from all causes). Based on these data, the American Academy of Pediatrics Committee on Injury and Poison Prevention developed a policy statement recommending that all children and adolescents use helmets while riding a bicycle and that pediatricians provide anticipatory guidance to facilitate universal helmet use (American Academy of Pediatrics and Committee on Injury and Poison Prevention 2001).

Despite apparent benefit, helmet use in children continues to be low with resultant increases in morbidity and mortality. Minnesota does not have a statewide law to compel the use of helmets, nor is there a county or city ordinance requiring helmet use in Olmsted County or Rochester, Minnesota (MN). This study was designed to investigate the incidence of bicycle-related injuries in Olmsted County among children ages 5 to 18 years between January 1, 2002, and December 31, 2011, enumerate the location and types of injuries, and analyze the differences in injuries between those children wearing and not wearing bicycle helmets.

## Methods

### Data source

Olmsted County, located in southeast Minnesota, is relatively isolated from other urban centers, rendering it amenable to epidemiologic research. The county consists of urban, suburban, and rural communities and has a population of 144,248 persons, of whom 86.5 % are white, 51.1 % are female, 24.9 % are under the age of 18 years, 94 % have graduated from high school or pursued a higher degree, and 8.5 % fall below the federal poverty level; the median household income is 66,667 dollars annually.

Local medical providers, consisting primarily of Mayo Clinic and its two affiliated hospitals, Olmsted Medical Center (OMC) and its affiliated hospital and the Rochester Family Medicine Clinic, deliver nearly all health care to the county population. All three institutions participate in the Rochester Epidemiology Project (REP), an infrastructure that permits the sharing of patient medical records to support population-based research (National Institute on Aging of the National Institutes of Health R01AG034676). The REP database comprises the records of all persons living in Olmsted County, MN. REP medical records include the details of every inpatient hospitalization at the three affiliated area hospitals; every outpatient visit to an office, clinic, urgent care, or emergency department; as well as every laboratory result and correspondence concerning each patient, and all individual facility records are linked to a single REP identification number for each Olmsted County resident. These records are accessible and easily retrievable because the REP has created extensive indices based on clinical and histological diagnoses and surgical procedures dating to the early 1900s.

### Identification of study subjects

Utilizing the REP electronic indices, all residents of Olmsted County between the ages of 5 and 18 years who had a discharge, primary, or secondary diagnosis of crash involving a bicycle between January 1, 2002, and December 31, 2011, were identified. Individual records were then retrospectively reviewed. Any subject using an alternative form of transport (e.g., moped, motorized bicycle, scooter) or riding as bicycle passenger was subsequently excluded from the study. Similarly, those children and adolescents who had been seen for another primary indication, but had been noted incidentally in the chart to have had a bicycle crash without further evaluation, were also excluded. Mayo Clinic Institutional Review Board (IRB), Olmsted Medical Center IRB, and REP approval, as well as a University of Minnesota IRB Authorization Agreement were obtained.

### Abstraction and tabulation of outcomes

Once identified, patients’ records were reviewed and data were collected including date of birth, sex, street address, and zip code; date of injury; circumstance of injury and potential associated risk factors (organized event—race/competition, cell phone use—call/text, impaired operator, or other (with free text box)); helmet use; date and time of acute care visit; mode of transport to medical facility; location and type of injury; Glasgow Coma Score (GCS); modality of diagnostic imaging performed and results; occurrence of trauma-related seizure; presence of residual permanent neurologic injury; need for inpatient hospital admission, pediatric intensive care unit (PICU) admission, and lengths of stay; need for mechanical ventilation, operative procedure, or discharge to a long-term rehabilitation care unit; and mortality. We defined a brain injury as a radiologically apparent sub-calvarial injury impacting brain parenchyma including epidural hematomas, subdural hematomas, subarachnoid hemorrhages, intraparenchymal bleeding/contusions, penetrating trauma, diffuse white matter injury, cerebral edema, or herniation; head injuries included the more superficial injuries of fractures, contusions, abrasions, and/or lacerations of the bony or soft tissues of the head. All data were tabulated into the REDCap database (an electronic data capture software designed by Vanderbilt University, allowing for secure, web-based management of online surveys and databases).

### Statistical analysis

Crash characteristics are summarized using mean ± standard deviation (SD) for continuous variables and frequency percentages for categorical variables. In order to calculate incidence rates, the identified cases were allocated to calendar period, age- and sex-specific categories for the numerator with the corresponding Olmsted County population counts used as the denominator (St Sauver et al. 2011). Age- and sex-adjusted incidence rates were directly standardized to the age and sex distribution of the 2000 US white population, a measure utilized given the racial homogeneity of Olmsted County. The calculation of standard errors and 95 % confidence intervals for the estimated incidence rates was based on the Poisson error distribution. Among cases that involved head injuries, the injury types and outcomes were summarized according to documented helmet use and compared between those documented to be wearing a helmet and those not wearing a helmet. Type of injuries, the percentage requiring head CT or X-ray, and hospitalization were compared using a chi-square test. PICU admission, permanent neurologic injury, seizure, need for mechanical ventilation, and mortality were compared using Fisher’s exact test. For these comparisons, two-tailed *p* values are reported with values ≤0.05 considered statistically significant.

## Results

Between January 1, 2002, and December 31, 2011, there were a total of 1189 acute care visits for bicycle-related injuries among Olmsted County residents 5 to 18 years of age. For these 1189 cases, the mean ± SD patient age was 10.5 ± 3.5 years; 882 (69.1 %) were male and 367 (30.9 %) female. Patient characteristics and circumstances of the bicycle-related crashes are presented in Table 1. Most occurred during the summer and the majority (86.3 %) involved a single bicycle. Five hundred (42.0 %) patients suffered head injuries, of which 97 (8.2 % of all crashes; 19.4 % of crashes involving head injury) resulted in an injury to the brain.

**Table 1:** Characteristics of 1189 bicycle-related injuries in Olmsted County, MN, 2002–2011

Characteristic	No. (%)
Age, years	
5 to 9	488 (41.0)
10 to 14	535 (45.0)
15 to 18	166 (14.0)
Sex	
Female	367 (30.9)
Male	822 (69.1)
Circumstances	
Single bicycle	1026 (86.3)
Bicycle vs. car	91 (7.6)
Bicycle vs. pedestrian	34 (2.9)
Bicycle vs. bicycle	9 (0.8)
Other	29 (2.4)
Location of injuries^a^	
Extremity	895 (75.3)
Head	500 (42.0)
Brain	97 (8.2)
Abdomen	50 (4.2)
Chest	41 (3.4)
Neck	14 (1.2)
Month of year	
January	2 (0.2)
February	4 (0.3)
March	28 (2.3)
April	102 (8.6)
May	162 (13.6)
June	202 (17.0)
July	208 (17.5)
August	221 (18.6)
September	165 (13.9)
October	73 (6.1)
November	19 (1.6)
December	3 (0.3)
Day of week	
Sunday	182 (15.3)
Monday	201 (16.9)
Tuesday	163 (13.7)
Wednesday	165 (13.9)
Thursday	155 (13.0)
Friday	146 (12.2)
Saturday	177 (14.9)

^a^Some patients had multiple injury locations.

### Incidence of bicycle-related injuries

**Fig. 1:**
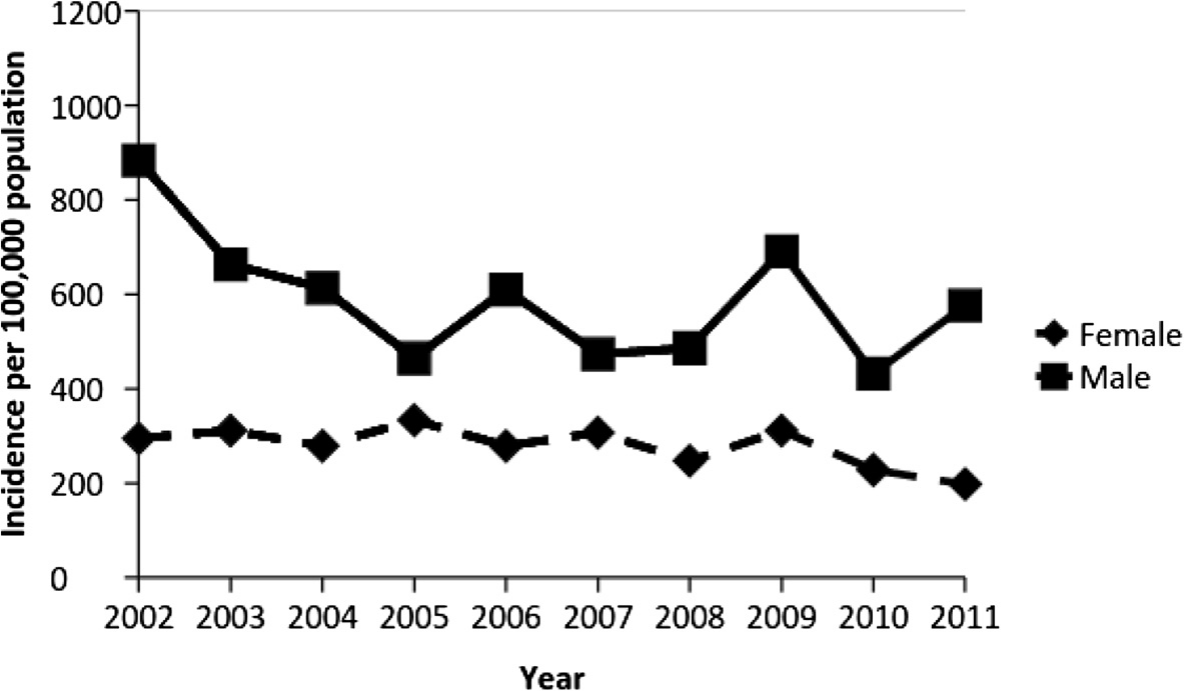
Age- and Sex-adjusted incidence of bicycle related injuries according to calendar year

For both male and female youth, the age-adjusted incidence of all bicycle-related injuries remained relatively consistent over the 10-year calendar period (Fig. 1). Table 2 presents the sex-specific and overall incidence rates for all injuries, head injuries, and brain injuries for various age groups.

**Table 2:** Incidence of bicycle-related injuries in Olmsted County, MN, 2002-2011

	Females			Males			Total		
Age group	No.	Rate^a^	95 % CI^a^	No.	Rate^a^	95 % CI^a^	No.	Rate^b^	95 % CI^b^
Any injury									
5–9 years	193	403	(346, 460)	295	585	(518, 652)	488	496	(452, 540)
10–14 years	150	327	(275, 380)	385	780	(702, 858)	535	560	(512, 607)
15–18 years	24	61	(36, 85)	142	352	(294, 409)	166	211	(179, 243)
All ages	367	278	(249, 306)	822	589	(549, 629)	1189	438	(413, 463)
Head injury									
5–9 years	89	184	(145, 222)	173	337	(286, 387)	262	262	(230, 294)
10–14 years	43	94	(66, 122)	132	267	(222, 313)	175	183	(156, 210)
15–18 years	7	18	(5, 31)	56	138	(102, 174)	63	80	(60, 100)
All ages	139	104	(87, 121)	361	255	(229, 281)	500	182	(166, 197)
Brain injury									
5–9 years	11	23	(9, 36)	18	35	(19, 51)	29	29	(18, 39)
10–14 years	13	28	(13, 44)	34	69	(46, 92)	47	49	(35, 63)
15–18 years	2	5	(0, 12)	19	47	(26, 68)	21	27	(15, 38)
All ages	26	20	(12, 27)	71	50	(39, 62)	97	35	(28, 43)

^a^Incidence per 100,000 person-years, age-adjusted to the US white population in the year 2000

^b^Incidence per 100,000 person-years, age- and sex-adjusted to the US white population in the year 2000

**Fig. 2:**
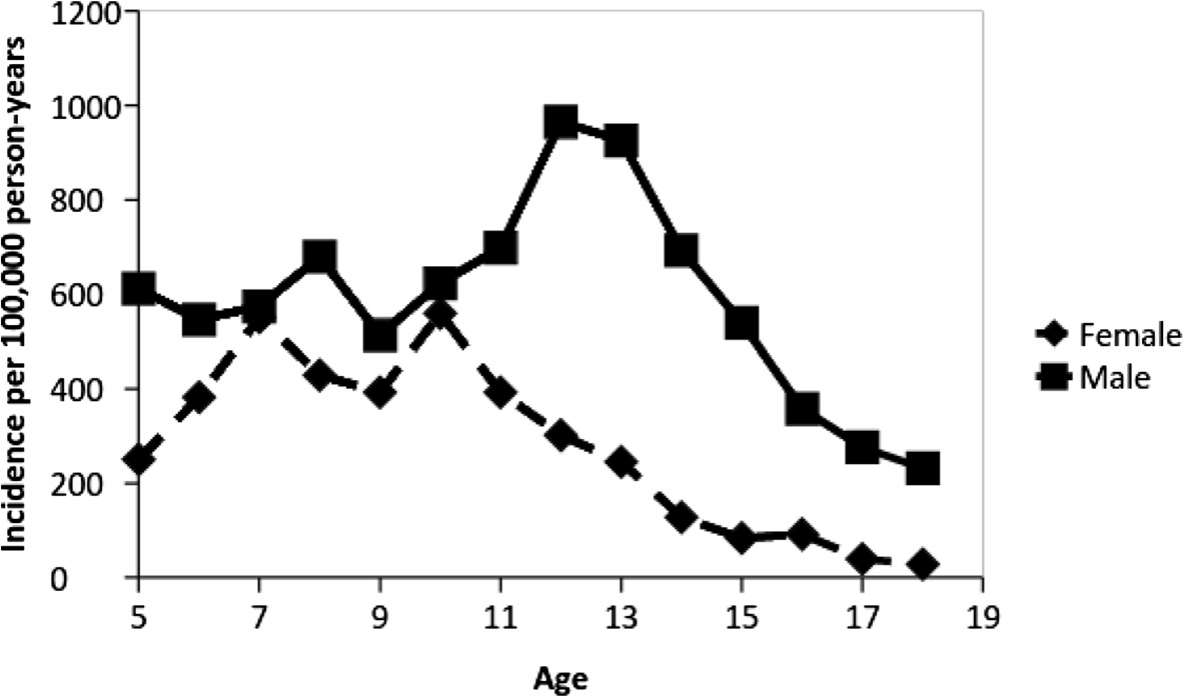
Incidence of bicycle related injuries according to age

**Fig. 3:**
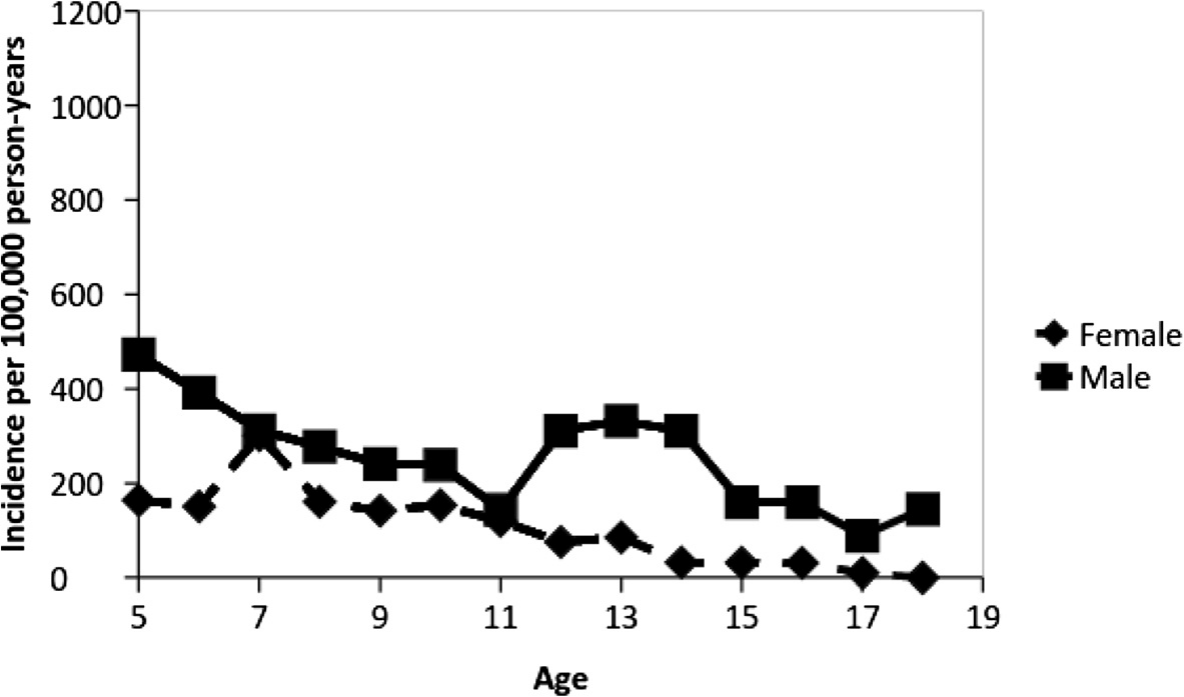
Incidence of bicycle related head injuries according to age

Reported previously, the incidence of bicycle-related injuries was lower for females compared to males. The overall age-adjusted incidence rate of all bicycle-related injuries was 278 (95 % CI, 249 to 306) per 100,000 person-years for females and 589 (95 % CI, 549 to 629) for males. The corresponding rates for head injuries were 104 (95 % CI, 87 to 121) per 100,000 person-years for females and 255 (95 % CI, 229 to 281) for males. The corresponding rates for brain injuries were 20 (95 % CI, 12 to 27) per 100,000 person-years for females and 50 (95 % CI, 39 to 62) for males. Figure 2 presents the sex-specific rates of all bicycle-related injuries for each year of age. The rate of all injuries in females remained relatively stable through age 10 years, after which it declined. The rate of all bicycle injuries in males remained relatively stable through age 10 years, after which it increased until the age of 13 years and then declined with increasing age. The sex-specific rates of bicycle-related head injuries for each year of age are presented in Fig. 3. For both females and males, the incidence of head injuries declined with increasing age.

### Helmet use

Of the 1189 bicycle-related injuries, 184 (15.5 %) were documented as wearing a helmet, 383 (32.2 %) were documented as not wearing a helmet, and 622 (52.3 %) had no documentation regarding helmet use. Compared to those without head injuries, patients with head injuries were significantly (*p* < 0.001) more likely to have documentation available regarding helmet use, with significantly (*p* < 0.001) more documented as not wearing a helmet. Of the 500 patients with head injuries, 17.4 % were wearing a helmet, 44.8 % were not wearing a helmet, and 37.8 % had no documentation regarding helmet use. Among the 689 patients without head injuries, the corresponding percentages were 14.1, 23.1, and 62.8, respectively.

Table 3 presents the type of injuries and outcomes according to helmet use for the 500 patients who suffered head injuries.

**Table 3:** Injury types and outcomes for those experiencing head injuries according to helmet use

	Helmet use			
	Yes (*N* = 87)	No (*N* = 224)	Unknown (*N* = 189)	
Characteristic	No. (%)	No. (%)	No. (%)	*p* value†
Type of injury^ab^				
Laceration	43 (49.4)	90 (40.2)	93 (49.2)	0.139
Contusion	29 (33.3)	91 (40.6)	52 (27.5)	0.236
Abrasion	41 (47.1)	123 (54.9)	80 (42.3)	0.217
Fracture	8 (9.2)	15 (6.7)	4 (14.8)	0.445
Brain injury	12 (13.8)	63 (28.1)	22 (11.6)	0.008
Other	17 (19.5)	46 (20.5)	33 (17.5)	0.845
Outcomes				
Head CT/X-ray obtained^b^	10 (11.5)	72 (32.1)	28 (14.8)	<0.001
Hospitalization^b^	3 (3.4)	19 (8.5)	5 (2.6)	0.144
PICU admission^c^	0 (0.0)	6 (2.7)	0 (0.0)	0.191
Permanent neurologic injury^c^	0 (0.0)	3 (1.3)	1 (0.5)	0.562
Post traumatic seizure^c^	0 (0.0)	2 (0.9)	1 (0.5)	>0.99
Need for mechanical ventilation^c^	0 (0.0)	3 (1.3)	0 (0.0)	0.562
Mortality^c^	0 (0.0)	2 (0.9)	0 (0.0)	>0.99

†Chi-square or Fisher’s exact test comparing the frequency of the given event between those wearing a helmet vs. not. Those with unknown helmet use are excluded from this comparison

^a^Patients may have had multiple injury types or experienced multiple outcomes

^b^Comparison performed using chi-square test

^c^Comparison performed using Fisher’s exact test

Compared to those wearing a helmet, those who were not wearing a helmet were significantly more likely to undergo an X-ray or computed tomography (CT) scan of the head (32.1 vs. 11.5 %; *p* < 0.001) and significantly more likely to experience a brain injury (28.1 vs. 13.8 %; *p* = 0.008).

### Severe outcomes

We identified 15 children and adolescents who suffered the most severe injuries, defined as those whose injuries required PICU admission or resulted in death. Of the 500 patients who suffered head injuries, 11 required PICU admission or succumbed to their injuries. Of these 11 patients, 10 were not wearing a helmet and 1 had no documentation regarding helmet use.

Two of these 11 suffered fatal injuries, an 18-year-old male and a 17-year-old female. The 18-year-old male was struck by a motor vehicle and had an initial GCS of 4. He underwent endotracheal intubation at the scene, was transported by helicopter to the hospital, and admitted to the PICU, where he subsequently died secondary to a severe traumatic brain injury (subdural hematoma and transtentorial herniation). The 17-year-old female, also struck by a motor vehicle, arrived in the emergency department with an absent pulse, a GCS of 3, and fixed and dilated pupils. Neither of these adolescents was wearing a helmet.

Of the remaining nine subjects with head and/or facial injuries, eight were not wearing a helmet. Helmet use was not documented for one patient. All nine suffered brain injuries (including one with temporal lobe hemorrhagic contusion), six suffered skull fractures (including two with epidural hematomas and two with pneumoencephaly), five were admitted to the PICU, three had seizures, and two suffered permanent neurologic damage. None required mechanical ventilation or discharge to a long-term rehabilitation care unit.

Four patients admitted to the PICU with bicycle-related injuries were admitted for injuries that did not involve the head and/or face, including two patients with liver lacerations, one patient with a laceration of the spleen who was also observed for risk of a cervical spine injury, and one patient with a femur fracture who required an operative procedure.

## Results and discussion

Our study aimed to estimate the incidence of bicycle-related injuries in children, describe the types and location of injuries, and analyze the differences in injuries between those children wearing and not wearing helmets in a county with no helmet ordinance. The incidence of all injuries and head injuries has remained stable over the 10-year period studied. Bicycle-related injuries were more common among males, in the summer months, and were predominantly single-bicycle events. Children and adolescents presenting with head injuries were more likely to have documentation of helmet use/non-use in the medical record and were more likely to have not been wearing a helmet. Among patients with head injuries, those not wearing a helmet were more likely to undergo X-ray or CT scan of the head and more likely to have suffered a brain injury.

The overall age-adjusted incidence rate of all bicycle-related injuries in our study ranged from 211 per 100,000 to 560 per 100,000 person-years; this rate is only slightly lower than the range of 392 per 100,000 to 663 per 100,000 children 18 years and younger found by Mehan et al. in a recent retrospective analysis of the National Electronic Injury Surveillance System (Mehan et al. 2009). Mehan et al. and many others, however, have published epidemiologic findings that are consistent with those of this study. For example, several studies demonstrate the higher likelihood of bicycle injuries among males (Thompson et al. 1990; Gallagher et al. 1984; Mehan et al. 2009). Bicycle-related injuries are also noted to increase with age, peak in middle childhood (with a slightly earlier peak for females), decrease in adolescence (particularly for females) (Thompson et al. 1990; Centers for Disease Control and Prevention 2004; Mehan et al. 2009), and, similar to our study, occur most often among males 10 to 14 years of age and least often among females 15 to 18 years of age (Mehan et al. 2009).

The decrease in bicycle-related injuries among adolescents, particularly females, has been noted previously (Mehan et al. 2009) and is perhaps related to overall lower bicycle-riding rates among adolescents (Evenson et al. 2003). Studies examining bicycling as a means of transportation to school have revealed that high school students are less likely than middle school students to bicycle to school (Evenson et al. 2003); additionally, distance to school (Timperio et al. 2006) (which may be greater for students in high schools as compared to elementary or middle schools) and car use (Lorenc et al. 2008) may play a role in adolescent bicycling. One researcher highlights the importance of lack of confidence in their ability to exercise correctly and a greater fear of injury among adolescent females as deterrents to bicycle riding (PedalTown Media, Inc. 2013).

Seasonal timing of bicycle injuries throughout the year is to some extent similar to what has been described previously in the literature, with a higher incidence in late summer in Olmsted County, MN, as compared to late spring and early summer in other geographic locations, likely reflective of weather conditions (Thompson et al. 1990).

Several studies have reported the effectiveness of bicycle helmets in reducing the risk of head, brain, and severe brain injury by 63 to 88 % (Thompson et al. 2000; American Academy of Pediatrics & Committee on Injury and Poison Prevention 2001; Mattei et al. 2012; Yeung et al. 2009) and facial trauma by 65 % (Thompson et al. 2000). Our study supports the higher likelihood of brain injury among children not using bicycle helmets. Indeed, of the 11 patients with the most severe outcomes, including two deaths, 10 were not wearing a helmet. Additionally, patients not using a helmet were more likely to have undergone diagnostic imaging. Webman et al. (2013) also found that non-helmeted cyclists were more likely to have had a CT scan of the head (42.1 %) compared to helmeted cyclists (31.0 %, *p* = 0.049). Similarly, a study of 108 cyclists, none of whom wore a helmet, performed by Munivenkatappa et al. (2013) found that 90 % of subjects underwent CT scan of the head. This finding is significant as it indicates an opportunity to potentially prevent unnecessary exposure to radiation and, perhaps, reduce healthcare costs.

In our study, helmet documentation was noted in 47.7 % of medical records reviewed. Compared to those without head injuries, patients with head injuries were significantly more likely to have documentation available regarding helmet use, with more noted to not have been wearing a helmet. Though studies have reported that children with serious head and brain injuries are less likely to have used a helmet (Maimaris et al. 1994), to our knowledge, no study to date has evaluated the likelihood of helmet documentation according to location of injury. Moll et al. found that helmet use was documented in only 23 % of 268 free text emergency department medical records of children ages 5 to 18 years (Moll et al. 2002). To address such issue, Monroe et al. demonstrated a statistically significant improvement in helmet use documentation from 49 to 77 % of emergency department records after implementing an injury documentation reminder sheet (Monroe et al. 2008). Standardization of helmet documentation in emergency department trauma records will not only improve availability of data but may also prompt a discussion with families at an opportune moment.

Efforts to attain universal bicycle helmet use have included educational campaigns, provider counseling, and mandatory helmet legislation. Education has involved promotion of helmet use or general safety information related to safe riding habits. A study evaluating the effect of a bicycle safety video revealed a significant improvement in retention of safety knowledge both immediately and 1 month later (Clements 2005). These studies suggest that that educational programs may both increase helmet use as well as decrease head injury rates (Rivara et al. 1998).

In one systematic review, helmet use was noted to increase from 15 % to over 50 % after educational intervention, although results were not equal across all socioeconomic strata, with low-income families demonstrating the least effect of the educational intervention (Clements 2005). Improving bicycle helmet use among economically disadvantaged children remains challenging.

Although helmet subsidies offered to low-income students increased helmet ownership from 10 to 47 %, observed helmet use was not different from that of three low-income control areas (18 vs. 19 %), while helmet use in higher income areas has been found to be as high as 73 % even prior to passage of mandatory helmet legislation (Parkin et al. 2003).

The American Academy of Pediatrics recommends that all pediatricians discuss bicycle helmet use as a part of anticipatory guidance (American Academy of Pediatrics & Committee on Injury and Poison Prevention 2001). One randomized, controlled trial (RCT) studied the effect of a single, brief counseling session on bicycle helmet use when given to injured adolescents in the emergency department setting; this study found that the intervention group was twice as likely to use a helmet 6 months following counseling relative to controls (Johnston et al. 2002).

Twenty-one states and the District of Columbia mandate bicycle helmet use for children, and many cities have local ordinances that require bicycle helmets for some or all riders (Insurance Institute for Highway Safety & Highway Loss Data Institute 2014). Mandatory helmet legislation is a factor strongly associated with helmet use by young children (American Academy of Pediatrics and Committee on Injury and Poison Prevention 2001). A recent Cochrane review found “positive evidence that bicycle helmet legislation both increases bicycle helmet use and reduces bicycle related head injuries (Macpherson and Spinks 2008)”. Furthermore, concern has been raised that bicycle helmet laws may result in decreased bicycle riding (Robinson 1996), with decreased physical activity as an unintended consequence. This possibility is of particular significance given the rising rates of pediatric obesity and overweight in the USA; unfortunately, data in the literature that address this possibility are scant and have limited capacity to support or refute this concern.

There are several limitations to this study. Helmet use was documented in only 47.7 % of medical records, making conclusions regarding the protective effect of helmets difficult; in addition, data regarding appropriate helmet size and use are not available. Importantly, any bicycle-related injury occurring during this time period that did not present to an acute care setting will not be captured. This would affect the calculated incidence and may bias our study toward capturing more severe injuries, and the proportion of severe to minor injuries, such as concussions, is not known. Furthermore, the REP does not represent hospital catchment area, and some patients may have presented to a facility outside of Olmsted County, particularly those patients with relatively trivial injuries at county borders.

As described, the Olmsted County population is largely white, educated, and of middle class income with a low poverty rate; hence, our results may only be generalizable to communities of similar populations. Notably, however, evidence suggests that children of families with middle income are more likely to wear helmets than those of families of low income. Furthermore, legislation may increase helmet use among these children to match, or potentially supercede, that among children of families with higher income, which may predict the effect of mandatory legislation in this county (Parkin et al. 2003).

## Conclusions

Children and adolescents continue to ride bicycles without wearing helmets, resulting in severe head and facial injuries and mortality. Educational campaigns, provider counseling, and mandatory helmet legislation may encourage helmet use and reduce bicycle-related head injuries.

## Abbreviations

CT: computed tomography
ED: emergency department
GCS: Glasgow Coma Score
PICU: pediatric intensive care unit
REP: Rochester Epidemiology Project
